# The experiences of women of reproductive age regarding health-promoting behaviours: A qualitative study

**DOI:** 10.1186/1471-2458-12-573

**Published:** 2012-07-30

**Authors:** Azam Baheiraei, Mojgan Mirghafourvand, Eesa Mohammadi, Sakineh Mohammad-Alizadeh Charandabi

**Affiliations:** 1 Department of Reproductive Health, Tehran University of Medical Sciences, Tehran, Iran; 2 Center for Community-Based Participatory Research, Tehran University of Medical Sciences, Tehran, Iran; 3 Midwifery Department, Tabriz University of Medical Sciences, Tabriz, Iran; 4 Department of Nursing, Tarbiat Modares University, Tehran, Iran

**Keywords:** Experience of health promoting behaviours, Women of reproductive age, Qualitative study

## Abstract

**Background:**

Health promotion is critical for community and family health. Health-promoting behaviours provide solutions for maintaining and promoting health. Although several studies have addressed the frequency and different types of health-promoting behaviours in women, little information is available about their experiences. This study aimed to explore the experiences of women of reproductive age regarding health-promoting behaviours.

**Methods:**

In the present study, which was conducted in Tehran, Iran, 15 females, who were selected purposefully, participated in individual in-depth, semi-structured interviews. The interviews were recorded, transcribed verbatim, and analysed using conventional content analysis.

**Results:**

Nine main categories were derived from the analysis, including establishing an appropriate eating pattern, establishing a balanced rest/activity pattern, spirituality, stress management, personal sensitivity and responsibility, establishing an appropriate pattern of social interactions, practicing safe and healthy recreations, feeling improvement in physical-functional health, and feeling improvement in emotional and psychological health. The first 7 categories represent the nature and types of real health-promoting behaviours in women of reproductive age, whereas the last 2 constitute feeling and understanding of the implementation of these behaviours.

**Conclusion:**

The study findings show that the women experience improvement in physical-functional, emotional, and psychological health by implementing health-promoting behaviours. It is therefore necessary to introduce strategies in the context of the community culture for improving different aspects of health-promoting behaviours in women of reproductive age to maintain and improve their overall health.

## Background

Health-promoting activities are strategies for maintaining health. A health-promoting lifestyle, as described by the World Health Organization [1], emphasizes the goal of ‘health for all’. A health-promoting lifestyle has considerable effects on increasing life span, improving the quality of life, and reducing health care costs [2]. Promoting healthy behaviours is the major concern of health care providers in the future decades [3].

Women’s health is the fundamental component of development and improvement in the developing world [4]. Prioritising women’s health will help to achieve the fourth and fifth goals of the millennium development program. The United Nations Millennium Development Declaration that includes 8 goals, signed in September 2000, commits world leaders to combat poverty, hunger, disease, illiteracy, environmental degradation, and discrimination against women [5]. Women nurture healthy individuals who are the centre of sustainable development; therefore, not paying attention to their health can lead to constant problems in the lifestyle and health of future generations [6]. Most Iranian women are in the reproductive age (60%), among whom 18% live in Tehran [7].

In previous studies, the concept of health-promoting behaviours in African–American females with faith-based support [8], the concept of health-preserving behaviours in the rural elderly of North Carolina [9], healthy behaviours and the associated factors among students of different cultures [10], and the type and spectrum of healthy behaviours among Chinese people with chronic diseases [11] were evaluated. In these studies, there were differences among different cultures and participants with regard to experiencing healthy behaviour. The culture and the value system in which a woman lives and her outlook and experiences about health-promoting behaviours affect health. Furthermore, each community has its own value system. Consequently, and in order to recognize the meaning of health-promoting behaviours from women’s viewpoint, it is necessary to conduct qualitative studies to develop the health services and interventions they need, and improve the performance of the health service providing system.

Qualitative studies are effective in assessing deep and personal concepts such as health promoting behaviours. This type of study helps to realize human phenomena by emphasizing its social context. By understanding women’s experiences of health-promoting behaviours, we can establish necessary plans for appropriate effective interventions in order to improve their health. The high number of women of reproductive age in Iran, their important role in family and community health, and the lack of qualitative studies about the concept of health-promoting behaviours from the women’s viewpoint led to the design of this qualitative study, aiming to explore women’s experience regarding health-promoting behaviours.

## Methods

### Participant selection and data collection

The present qualitative study aimed to explore the experiences of women of reproductive age about health-promoting behaviours. This method is able to clarify people’s understanding of daily life [12]. This study was conducted in Tehran (capital of Iran) in 2011.

Iran is a diverse country with a number of ethnic groups. The Iranian economy is the second largest in the East Mediterranean Region after Saudi Arabia, with a gross domestic product of over US$ 200 billion [13]. Tehran is the most populous city of Iran, with 12 million inhabitants including 3.8 million women of reproductive age [7]. This study is the qualitative part of a mixed-method study using a sequential explanatory design including 2 phases, the protocol of which has been previously published [14]. Participants of the present study were selected purposefully in the form of extreme cases sampling on the basis of the results of the quantitative phase. The women whose health promoting behaviour scores were in the 10th and the 90th percentiles and who could express their experiences about health-promoting behaviours were selected for interviews in the qualitative phase.

The participants’ demographic data are shown in Table 1. Sampling was continued until data saturation was achieved—that is, no new information or codes were present in the data— which happened at the 12th interview. A total of 15 women participated in the interviews. Interviews ranged from 20 to 120 minutes in length. The corresponding author conducted individual in-depth, semi-structured interviews. In-depth interviews are useful to explore the individuals’ understanding and experiences [15]. The interviews started using general open-ended questions like ‘1- What do you do to maintain or promote your health?’ Then, focusing on each of the expressed behaviours, we asked ‘2- How do you do it?’ and ‘3- How do you feel when you exercise this behaviour?’ Continuing the interview, according to the responses to each of the questions, in-depth probing questions such as ‘What do you mean?’ ‘Why?’ ‘Explain more’ and ‘Would you please give an example to better convey what you mean?’ were asked in order to find out the depth of the women’s experience. During the interviews, the researcher recorded nonverbal data such as the participants’ tone, facial expression, and position as well as the time and the location of the interview on a special sheet. The interviews were conducted at participants’ convenience in places such as a park, home, and university. 

**Table 1:** Sociodemographic characteristics of participants

**Characteristic**	**Number**	**Percent**
**Age (in years)**		
15–24	5	33.3
25–34	6	40
35 or above	4	26.7
**Education**		
Elementary & secondary school	2	13.3
Diploma	4	26.7
University	9	60
**Marital status**		
Single	10	66.7
Married	5	33.3
**Occupation**		
Housewife	6	40
Employed	4	26.7
Student	5	33.3
**Total**	15	100

### Data analysis

MAXQDA software version 10 was used for data management, and after each interview, the data were analysed using content analysis with a conventional approach. The advantage of a conventional approach in qualitative content analysis, as in the present study, is the ability to gather data directly from study participants without imposing pre-conceived categories and previous theoretical perspectives. In this method, knowledge generated from content analysis is based on the unique views of the participants and is rooted in text data [16]. The interviews, which were recorded on tapes, were transcribed verbatim. The transcribed interviews were read several times. The meaning units related to health promoting behaviours were identified. The codes detected on the basis of the meaning units resulted from the participants’ explanations. The codes were classified into main categories and subcategories on the basis of differences and similarities. Examples of the content analysis, encoding, subcategories, and main category are shown in Table 2. 

**Table 2:** An example of analysis process

**Meaning unit**	**Code**	**Sub Category**	**Main Category**
I believe and deeply think that the universe is God’s presence and we’d do better to sin less. This belief really helps me to find the right way.	Paying attention to being in God’s presence and responsibility	**Attending to and strengthening religious beliefs and attitudes**	**Spirituality**
I look for what makes God satisfied with me, I do the same thing.	Considering God’s satisfaction in the works		
Believing that what God puts in our way is all beneficial, is all God’s grace to humans, that is, believing that God will never expect bad for his creatures, this is really helpful in being happy and hoping for the future.	Having faith in the grace of God		
Oh God, I’m satisfied with your satisfaction, I say. If something happens to someone or she loses one of her loved ones, she must to be so strong so that she can say thank you God for doing so; one should not be impatient or complain then.	Being satisfied with God’s satisfaction		
What helps my spiritual health is my trust in God. Just in God I trust, and I tell myself always what God wants happens.	Trust in God		
My religion is important for me. I really appreciate it. My hijab isn't very well but I highly believe in faith, mysticism, and such things. Religious issues are very important to me.	Belief in religion and religious issues		
I believe the more knowledge one has about her religion the more she enjoys her life and is happy.	Knowing more about one’s religion is a reason to enjoy life.		

### Ethics

This study was approved by the Ethics Committee of Tehran University of Medical Sciences and informed written consent was obtained.

### Trustworthiness

To increase trustworthiness of the data, a rapport was established with the participants and enough time was allocated for the data collection. This study relied primarily on two types of data: transcripts generated from interviews and the interviewers’ field notes. Interview transcripts and the derived codes from each of the interviews were presented to the participants and their views about the nature and the meaning of the codes were asked; if they expressed opposing views, their corrective comments were applied. In addition to the study team, the text of the interviews was presented to some of the researchers who were not involved in the study as external observers and they were asked to check the accuracy of the coding process.

## Results

We reached data richness, saturation, and repetition by a total of 15 interviews with women of reproductive age. A total of 309 codes and 30 subcategories were extracted, which were classified into 9 main categories including establishing appropriate eating patterns, establishing a balanced rest/activity pattern, spirituality, stress management, personal sensitivity and responsibility, establishing an appropriate pattern of social interactions, practicing safe and healthy recreations, feeling improvement in physical and functional health, and feeling improvement in emotional and psychological health (Table 3).

**Table 3:** Classification of main categories and subcategories

**Main categories**	**Subcategories**
Establishing an appropriate eating pattern	- Dedication to balanced consumption of all food groups
	- Meals and appropriate pattern of eating
	- Proper cooking
	- Good food preparation and storage
Establishing a balanced rest/activity pattern	- Dedication to appropriate and favourable exercises
	- Enough rest/sleep
Spirituality	- Self-awareness and self-construction
	- Thinking and optimism
	- Adhering to the religious rituals
	- Attending to and strengthening religious beliefs and attitudes
Stress management	- Relaxation and making happy moments
	- Tolerance and compromising with problems
Personal sensitivity and responsibility	- Regular assessment of physical health
	- Observing medical recommendations and advice
	- Observing personal hygiene
Establishing an appropriate pattern of social interactions	- Participation in community service
	- Tendency and dedication to social integration
	- Close family relationships
	- Relationship with the children
Practicing safe and healthy recreation	- Travel and nature tours
	- Growing flowers and plants
	- Reading and painting
	- Watching television and listening to music
Feeling improvement in physical and functional health	- Sense of increased energy and physical strength
	- Improved sleep
	- Sense of being able to prevent disease occurrence and progression
Feeling improvement in emotional and psychological health	- Feeling joy and vitality
	- Reducing tension and creating relaxation
	- Feeling good understanding of self
	- Satisfaction and increased confidence
	- Enhanced thinking and positive thinking
	- Improving mood

### Establishing an appropriate eating pattern

Establishing an appropriate eating pattern is a main category which shows the broad and comprehensive dedication and attention of women to different aspects of nutrition. This category was derived from subcategories of dedication to balanced consumption of all food groups, meals, an appropriate pattern of eating, proper cooking, and good food preparation and storage.

One of the interesting aspects of nutrition for the participants was the type of food consumed during the day. For example, about the type of foods, 2 of the participants pointed out:

‘I try to fit healthy things such as milk, vegetables, fruits, and so forth in my diet.’ [p. 4]

‘I generally use anything that is good and useful for the health’. [p. 3]

Another feature of interest for women in establishing an appropriate eating pattern is meals; that is, women were trying to have an appropriate eating pattern to protect their own health. For example, they tried to take a full and regular breakfast; on the contrary, they had a small snack for dinner. For example, 2 of the participants expressed their meals as follows:

‘We try to eat dinner early because it both affects our health and helps us sleep comfortably.’ [p. 3]

‘I don’t eat dinner, but I have a complete breakfast.’ [p. 5]

Women considered correct and appropriate food cooking skills to be a factor affecting health. For example, about food cooking, one of the participants said:

‘This is a kind of art in my opinion. As one enjoys watching a movie, I myself enjoy cooking by mixing some raw materials and preparing a so-called eatable thing, which has a preserved quality, as well as an appropriate taste, seasoning, and colour. This is a kind of art, as well.’ [p. 2]

Another aspect of nutrition was food preparation and storage, which was mentioned by the participants. For example, a participant commented:

‘When I prepare food, I try to use healthy things. The nutrients must be healthy and fresh, as much as possible of course. If not possible, well I’ll use frozen materials, a little though.’[p. 6]

### Establishing a balanced rest/activity pattern

Establishing a balanced rest/activity pattern is the main category derived from the subcategories of women’s dedication to appropriate and favourable exercises and enough rest/sleep. The analysis of the data from the experiences of the participants suggested that they intended to make a balance between their activity and rest to maintain and improve their health.

The participants considered exercising as a health factor and exercised appropriate to their interest and beliefs or their age. For example, 2 of the participants said:

‘I started with jump roping; you know that during the years 1974–1975 (about 35 years ago) there were no facilities or clubs in small towns. Later on, during mid school, I learned ping-pong and then I was champion in my town. During high school, I didn’t do a special sport except that I was on the school volleyball team. A bike was always found at home, and I was always biking in my free time at home. After the Cultural Revolution, when I was at the university, I did not follow a specific sport either; however, I replaced my sport interests with Swedish exercise and jogging. Then, I continued these exercises, especially jogging.’ [p. 2]

‘I do taekwondo. I like to do it because it relaxes me.’ [p. 3]

Another point which women observed to maintain their health was having enough sleep and rest. For example, one of the participants stated:

‘I try to sleep earlier at night so that I am fresh when I’m supposed to wake up early in the morning. I try to take a shower when I come back home, relax and have more rest, sleep early, and wake up fresher.’ [p. 15]

### Spirituality

Another category derived from data analysis of the experiences of the participants was spirituality, which shows that spirituality is an important aspect of the participants’ lifestyle. This category was derived from the subcategories of self-awareness and self-construction, thinking and optimism, adhering to religious rituals, and attending to and strengthening religious beliefs and attitudes.

One of the aspects of spirituality the participants attended to in order to maintain and improve their health was the effort for self-awareness and self-construction, as 2 of the participants pointed out:

‘In order to achieve self-awareness, I analyse my thoughts and behaviour when my behaviour was ugly and bad, when I flew off the handle, when I did something with others which I shouldn’t have done at all.’[p. 3]

‘I always try to be straight up and honest.’[p. 2]

Positivism and optimism were other aspects of spirituality women tried to possess in order to maintain and improve their health. For example, one of the participants stated:

‘Positivism really helps me. I believe that a day, someone but not inevitably a hero- just a human being, will anyway present an idea which will have more positive effects than today, even if that day I'm not alive.’[p. 2]

Adhering to religious rituals, such as going to mosques and on pilgrimages and praying were further activities women used to improve their health. Two of the participants stated:

‘Yes, reading prayers and pilgrimages and such things are in my schedule. I read the Quran everyday which gives me a very pleasant feeling.’[p. 14]

‘I look to prophets and Imams for what they have said and try to follow their behaviour.’[p. 3]

Another aspect of the spirituality to which the participants were dedicated was strengthening religious attitudes and beliefs. One of the participants, for example, pointed out:

‘I believe and deeply think that God is omnipresent and we’d better sin less. This belief really helps me to find the right way.’ [p. 2]

### Stress management

Stress management is a main category which reflects the dedication of women to control stress. The analysis of the data from participants’ experiences shows that they used different techniques to control their stress in order to maintain and promote their health. This category was derived from subcategories of relaxation and making happy moments, and tolerance and compromising with problems.

One of the strategies women used to control their stress was to try to make the moments of their lives happy and relaxing using different techniques. For example, 2 of the participants stated:

‘Laughing, without exercising, helps my health.’[p. 2]

‘Considering spiritual health, I always try to think of good things and better situations.’[p. 1]

The second strategy that women used to control their stress and, as a result, promote their health was acceptance of and compromising with problems. Two of the participants pointed out:

‘I believe that I should accept the undesirable events in my life, everything that makes me stronger.’ [p. 2]

‘I try to cope with my problem, face it, and solve it.’[p. 3]

### Personal sensitivity and responsibility

Another main category derived from the analysis of the data from the participants’ experiences was personal sensitivity and responsibility, which shows that women felt responsible for their health. This category was derived from the subcategories of regular assessment of physical health, observing medical recommendations and advice, and observing personal hygiene. One of the actions the participants took to improve their health was regular assessment of physical health, as one of the participants expressed:

‘I have a full medical check-up every 6 months’. [p. 11]

Another action which shows personal sensitivity and responsibility of the participants was observing personal hygiene. For example, one of the participants stated

"‘I try to take care of hair hygiene and stuff. I brush and floss my teeth every night. I take a shower every other day for my hair hygiene. I also care for my skin and use sunscreens’. [p. 6]

### Establishing an appropriate pattern of social interactions

Establishing an appropriate pattern of social interactions is a main category which shows women’s attention to and awareness of the importance of social interactions and interpersonal relationships in improving, maintaining and promoting their health. This category includes the subcategories of participation in community services, tendency and dedication to social integration, and close family relationships and relationships with children. These all are the dimensions of women’s attention to create an appropriate pattern of social interactions.

One of the dimensions of social interactions which were considered by women was participation in community services. One of the women said the following about social interactions:

‘Another thing which also makes me happy to have a healthy life is to help people in need expecting nothing in return.’[p. 5]

Another dimension, which was considered by women to establish an appropriate pattern of social interaction was the tendency and dedication to social integration. For example, one of the participants stated:

‘I try to behave in a socially acceptable way. I want everybody to be satisfied with me. Well, this affects my health as well.’[p. 4]

Having close family relationships was another dimension of social interactions women considered in promoting their health. For example, one of the participants stated:

‘In order to improve my mental health, if my husband has enough time, we try to visit our parents and our families during the week.’[p. 13]

One of the participants considered building a good relationship with children as an important way of reducing unhappiness; she expressed her experience as:

‘When I'm bored or feel upset or don't have a good feeling or I'm really tired, I try to play with a couple of young children. I myself play the role of a child and during that time I don't even think of those problems that bothered me. I play in the playgrounds, parks, with relatives’ children. I don’t go anywhere else.’ [p. 5]

### Practicing safe and healthy recreations

Practicing safe and healthy recreations indicate attention of the women of reproductive age to having appropriate pastime activities to maintain and promote their health. This category was derived from the subcategories of travel and nature tours, growing flowers and plants, reading and painting, and watching television and listening to music. Travelling and going to the park, nature, and green environments were among the recreations that women took in order to maintain and promote their health. Two of the participants stated:

‘The thing I myself really like is to communicate with nature and animals.’ [p. 5]

‘We try to go to a park or have short trips to somewhere not so far and come back.’ [p. 13]

Flower and plant growing was another recreational behaviour women paid attention to, as one of the participants said:

‘One other thing I really enjoy is watering plants. This gives me a pleasant feeling because I talk with them.’[p. 5]

Reading different books and painting were among other healthy recreations women did to maintain and promote their health. For example, 2 of the participants stated:

‘Studying especially helps improve my health; it shows one the solutions… In my opinion, studying really helps people to at least think better, and when one thinks better, she naturally finds better solutions, as well. The horizon of human thought is not limited. The world of human thought is not small. These all help my physical and mental health.’[p. 2]

‘I paint for my mental health. Painting takes most of my time.’ [p. 9]

### Feeling improvement in physical and functional health

Feeling improvement in physical and functional health is a main category which shows the participants feel better in physical and functional dimensions of health by practicing health improving behaviours. This category was derived from the subcategories of a sense of increased energy and physical strength, improved sleep, and a sense of being able to prevent disease occurrence and progression.

The participants felt that having health-improving behaviours resulted in gaining and increasing energy and their physical strength. For example, one of the participants stated:

‘I feel that exercise provides me with energy for a long time. I get out of those sad moods. I really get bothered when I stay at home, but I feel energetic when I do exercises.’ [p. 11]

The participants understood the positive effect of having health-promoting behaviours on improving sleep status; for example, one of the participants said:

‘When I have a healthy diet, I get a good sleep.’ [p. 5]

The participants understood that practicing health-promoting behaviours prevents the occurrence and progression of diseases. For example, one of the participants pointed out:

‘When one goes for a medical check-up, she knows her health status, and prevents diseases if there is something; she observes herself. If there is something wrong in the lab tests or check-up exams, one can easily prevent it instead of letting it get piled up and end in an acute disease.’ [p. 1]

### Feeling improvement in emotional and psychological health

Participants felt an improvement in the emotional and psychological dimensions of their health, as well as physical and functional dimensions, when they were engaged in health promoting behaviours. This category was derived from the subcategories of feeling joy and vitality, reducing tension and creating relaxation, satisfaction and increased confidence, enhanced and positive thinking, and improving mood.

The participants stated health-promoting behaviours as one of the factors affecting their experiencing freshness, liveliness, and vitality. For example, one of the participants stated:

‘The habit of exercising is planted inside me! This sense of vitality is inside me and is one of the reasons why I’m a happy woman.’ [p. 2]

Women felt that doing health-promoting behaviours results in reduced tension and provides them with relaxation. So, one of the participants pointed out:

‘Our religious ceremonies really help me feel relaxed in my spiritual health. Attending the religious ceremonies and meetings results in cleaning up the spirit, feeling free, feeling closer to God, calming down, feeling confident about life’s concerns, having confidence in God for everything and strengthening the bond with God.’[p. 14]

One of the participants understood the positive value of exercise in feeling good understanding of oneself and expressed her feeling as:

‘Despite all the present problems and issues, exercise has taught me patience. I have a high self-confidence and appreciate the exercise time. I like to exercise all the time. Exercising has led me to know myself better.’[p. 2]

Women felt that doing health-promoting behaviours leads to increased self-confidence and a feeling of satisfaction and pleasure. As one of the participants pointed out:

‘When I behave well, I feel satisfied. When I know that I behaved the way I like, I have a feeling of satisfaction, power, self-confidence, and perfection. That is, when I go somewhere and come back without being upset, I feel that that day was perfect; I didn’t get unhappy, I mean, of my behaviour.’[p. 14]

The participants understood the positive effect of health-promoting behaviours on strengthening thought and positive thinking; for example, one of them said:

‘Participating in meditation classes makes me think positively and love everyone.’ [p. 5]

Women thought that health-promoting behaviours lead to improved mood; for example, one of the participants pointed out:

‘When I exercise, the energy it gives me makes me more modest in treating people or in my behaviours, ethical behaviours, and treating others.’[p. 1]

## Discussion

The present study is the first study in Iran to address women’s experiences of health-promoting behaviours. The results of this study show that women practice behaviours such as establishing appropriate eating patterns, establishing balanced rest/activity patterns, spirituality, stress management, personal sensitivity and responsibility, establishing appropriate patterns of social interaction, and practicing safe and healthy recreation in order to maintain and improve health. This study suggests that Iranian women perform a wide range of health-promoting behaviours to maintain and improve their health. The experiences of Iranian women with regard to health-promoting behaviours are derived from their culture, and their values are rooted in the context they live in. This interaction is inevitable and supported by a comparison of this study with other studies on health-promoting behaviour.

In another qualitative study, African–American women laid emphasis on the importance of exercise, a balanced diet, weight control, stress reduction, and annual health examination for health maintenance. The health-promoting behaviours that were important for these women included evaluation of serum lipids, maintaining a constant blood pressure, resting and exercising, walking, drinking fluids and water, healthy nutrition (including the use of simple food, healthy nutritional habits [such as eating halal meat, using vitamins, and avoiding fats and cookies]), and adopting a low-salt diet [8]. In a study conducted with students of different cultures, exercising regularly, adopting a proper diet, maintaining an ideal weight, sleeping for 6–8 hours every day, dealing effectively with stress and being emotionally healthy, not smoking, and drinking alcohol in moderation were considered beneficial for health by all groups; however, there were differences in some behaviours. For example, only Taiwanese students participated in religious activities [10].

Arcury et al. (2001) determined the following healthy behaviours from the participants’ responses: eating properly, drinking water, exercising, being with people, staying busy, believing in God and participating in church activities, and taking care of oneself [9]. Compared with the present study, there were differences in reported healthy behaviours. For example, the behaviours ‘being with people’, i.e. maintaining social contacts, and ‘staying busy’, i.e. performing joyful activities were different from the behaviours ‘establishing an appropriate pattern of social communications’ and ‘practicing safe and healthy recreation’ derived from our study. Additionally, stress management was not reported in the Arcury et al. study. In the present study, spirituality encompassed many aspects; however, Arcury et al. found that participants described only some aspects of spirituality, namely a belief in God and participating in church.

A behaviour that was reported by all qualitative studies [8-10] and the participants of the present study to be health-promoting behaviour was adopting healthy nutrition practices, which is also the main component of health promotion [17]. The participants paid attention to all aspects of an appropriate nutritional pattern, such as type of food, number of meals, processes of cooking, and food preparation and storage; however, in the previous studies, only 1 or 2 aspects of healthy nutrition were reported. This approach to health promotion will improve the quality of life and result in reduced health care costs [18].

Another health-promoting behaviour was physical activity, which was a consistent finding in all qualitative studies and was also found in the present study. The studies and surveys provided definitive evidence about several beneficial effects of increased physical activity, such as preventing premature death caused by coronary artery disease, type II diabetes, and colon cancer, especially in individuals with a sedentary lifestyle. Exercising also promotes mental health and reduces the risk of developing obesity and osteoporosis [19].

As described above, spirituality was more widely considered in the present study, and the aspects considered included self-awareness and self-construction, contemplation and optimism, adhering to religious rituals, and strengthening religious beliefs and attitudes. However, other studies did not report this aspect of behaviour or only addressed the aspects of adhering to religious rituals as well as strengthening religious beliefs and attitudes. The spiritual aspect is an important aspect of human function and positively influences health [20].

Stress management was another activity implemented by the Iranian women for maintaining and promoting health. As previously indicated, this aspect was considered in some qualitative studies. Stress can cause problems in physical and mental health [21] and can negatively influence the acceptance and performance of health-promoting behaviours [22]. As a result, stress management not only directly improves women’s health but also further enhances other health-promoting behaviours.

Personal sensitivity and responsibility was another category derived from the participants’ responses that showed that women consider health maintenance to be a personal commitment and duty. The participants’ responses indicated that they knew the importance of prevention and considered themselves responsible for their health. It is favourable for people to bear responsibility for their own health. Individuals who effectively protect their health are usually healthier, both mentally and physically [23].

Establishing an appropriate pattern of social communication, which is known as one of the most important social determinants of health [24], was another behaviour that was implemented by the Iranian women for maintaining and promoting their health. Although interpersonal relationships were addressed by some previous qualitative studies [9], various aspects including participation in community service, dedication to social integration, close family relationships, and relationships with children were considered in the present study. The present findings may be affected by the context of the Iranian culture, in which family members have close relationships with each other and there is a strong intra- and inter-group social network.

Practicing safe and healthy recreation was another behaviour that was practiced by most of the participants in order to maintain and improve their health. Recreation and hobbies are important parts of a healthy lifestyle. A previous study showed that participation in recreational activities resulted in the improvement of physical and social function as well as mental health [25].

The findings of the present study show that women experienced an improvement in physical-functional and mental health when they implemented different health-promoting behaviours, which are consistent with the findings of Lucas et al. (2000) who observed enhanced psychological and functional health in individuals participating in health-promoting activities, especially physical activity. Furthermore, these individuals reported that health-promoting behaviours helped them to adapt to the problems associated with aging and to manage health-specific problems. Moreover, these behaviours provided opportunities for social communication [26]. In another study, the participants indicated that a healthy diet led them to have more energy and made them feel healthier [3]. Shuster et al. (2009) showed that the women who performed more physical activities were healthier and had higher levels of physical and social function [27]. This finding indicates the importance of health-promoting behaviours in improving the main aspects of health.

A comparison of the results of the present study with those of other studies in this field in different cultures suggests that the behaviours applied in our community are different from those in other communities. It is therefore necessary to introduce strategies in the context of the community culture for improving different aspects of health-promoting behaviours in women of reproductive age to maintain and improve their overall health.

Similar to other qualitative studies, the findings of the present study have low potential for generalisation, especially because the present study was conducted on a limited number of women of reproductive age in Tehran. Moreover, most of the participants had a diploma or a university education; as a result, the findings should be cautiously generalised to other women.

## Conclusion

The findings of the present study show that women practice some behaviours, such as establishing an appropriate eating pattern, establishing a balanced rest/activity pattern, spirituality, stress management, and practicing safe and healthy recreation in order to maintain and improve their health. By performing these health-promoting behaviours, they feel an improvement in their physical-functional and mental health. Understanding the experiences of women of reproductive age regarding health-promoting behaviours can help researchers and healthcare providers to develop strategies to promote positive changes in lifestyle, to facilitate long-term behavioural changes in women, and to ultimately improve the health of women according to social and cultural conditions.

## Competing interests

The authors declare that they have no competing interests.

## Authors’ contributions

All the authors contributed to the conception and design of the study. MM wrote the first draft of the paper. AB, EM, SN, and SM revised the manuscript. AB reviewed the manuscript for important intellectual content. All authors read and approved the final manuscript.

## Pre-publication history

The pre-publication history for this paper can be accessed here:

http://www.biomedcentral.com/1471-2458/12/573/prepub
